# The Effect of COVID-19 Vaccination on the Risk of Persistent Post–COVID-19 Condition: Cohort Study

**DOI:** 10.1093/infdis/jiaf133

**Published:** 2025-03-12

**Authors:** Pontus Hedberg, Suzanne Desirée van der Werff, Pontus Nauclér

**Affiliations:** Department of Medicine Huddinge, Karolinska Institutet, Stockholm, Sweden; Division of Infectious Diseases, Department of Medicine, Karolinska Institutet, Stockholm, Sweden; Department of Infectious Diseases, Karolinska University Hospital, Stockholm, Sweden; Division of Infectious Diseases, Department of Medicine, Karolinska Institutet, Stockholm, Sweden; Department of Infectious Diseases, Karolinska University Hospital, Stockholm, Sweden

**Keywords:** COVID-19, SARS-CoV-2, post–COVID-19 condition, long COVID, Vaccination

## Abstract

We conducted a population-based cohort study in Stockholm, Sweden, to investigate the effect of COVID-19 vaccination on the risk of developing persistent post–COVID-19 condition (PCC) in individuals surviving the first year after a SARS-CoV-2 infection. In total, 331 042 individuals were included, of which 852 had persistent PCC. The adjusted risk ratio for developing persistent PCC compared with unvaccinated individuals was 0.81 (95% confidence interval [CI], .59–1.10) for 1 dose, 0.42 (95% CI, .35–.52) for 2 doses, and 0.37 (95% CI, .27–.52) for 3 doses. Reduced risks for vaccinated individuals were also observed when restricting the analyses to pre-Omicron and Omicron, as well as all subgroups including sex, age, and previous infection.

## Teaser

In this population-based cohort study set in Stockholm, Sweden, COVID-19 vaccinations were strongly associated with a reduced risk of persistent post–COVID-19 condition.

The natural history of post–coronavirus disease 2019 condition (PCC) is poorly understood [1]. We have previously shown that a PCC diagnosis correlates with excess health care usage in severe acute respiratory syndrome coronavirus 2 (SARS-CoV-2)–infected individuals, indicating a good accuracy of the diagnosis for severe PCC [2], and a Swedish cohort study found an association between coronavirus disease 2019 (COVID-19) vaccination and a reduced risk of being diagnosed with PCC [3]. Whether COVID-19 vaccination also influences the risk of experiencing long-term, persistent PCC remains to be understood. In this study, we aimed to investigate whether COVID-19 vaccination was associated with a reduced risk of persistent PCC.

## METHODS

We conducted a retrospective population-based cohort study in Stockholm County, Sweden. The study was approved by the Swedish Ethical Review Board (reference number, 2018/1030-31, COVID-19 research amendments reference number, 2020-01385, 2020-02145, 2020-04069, and 2022-02127-02). The need for consent was waived by the Swedish Ethical Review Authority because analyses are based on retrospectively collected data from the administrative health registry.

Data were linked from 5 population-based data sources: Stockholm regional health care data warehouse, SmiNet, Statistics Sweden, the Swedish Intensive Care Registry, the National Vaccination Register, and the Quality Register for SARS-CoV-2 (COVID-19), as previously described [2, 4]. The study population consisted of individuals aged ≥18 years with a polymerase chain reaction (PCR)-verified infection from 10 January 2021 (14 days after the first COVID-19 vaccine was administered in Sweden) until 9 February 2022 (last day of public testing) (Supplementary Figure 1). The exposure was COVID-19 vaccination status 14 days before the SARS-CoV-2–positive PCR test (unvaccinated, 1 dose, 2 doses, or 3 doses). Persistent PCC was defined as having a PCC diagnosis code (International Classification of Diseases-Tenth Revision [ICD-10] U09.9) registered at least once 90 to 364 days and once 365 to 660 days (longest possible follow-up for all individuals) after the PCR test. Individuals with less than 365 days of follow-up after the positive test were excluded. Individuals were censored at the date of death, date of moving out of Stockholm County, or 660 days after the positive test. Modified Poisson regression models adjusting for age, sex, region of birth, education level, comorbidities, previous infection, SARS-CoV-2 variant period, and days since 9 January 2021 were used to obtain risk ratios (RRs) with 95% confidence intervals (CIs) of developing persistent PCC. Subgroup analyses included pre-Omicron, Omicron, male sex, female sex, aged < 65 years, aged > 65 years, no verified previous infection, and verified previous infection. Study variables are described in Supplementary Table 1. Analyses were conducted with R version 4.1.0.

## RESULTS

**Figure 1.**
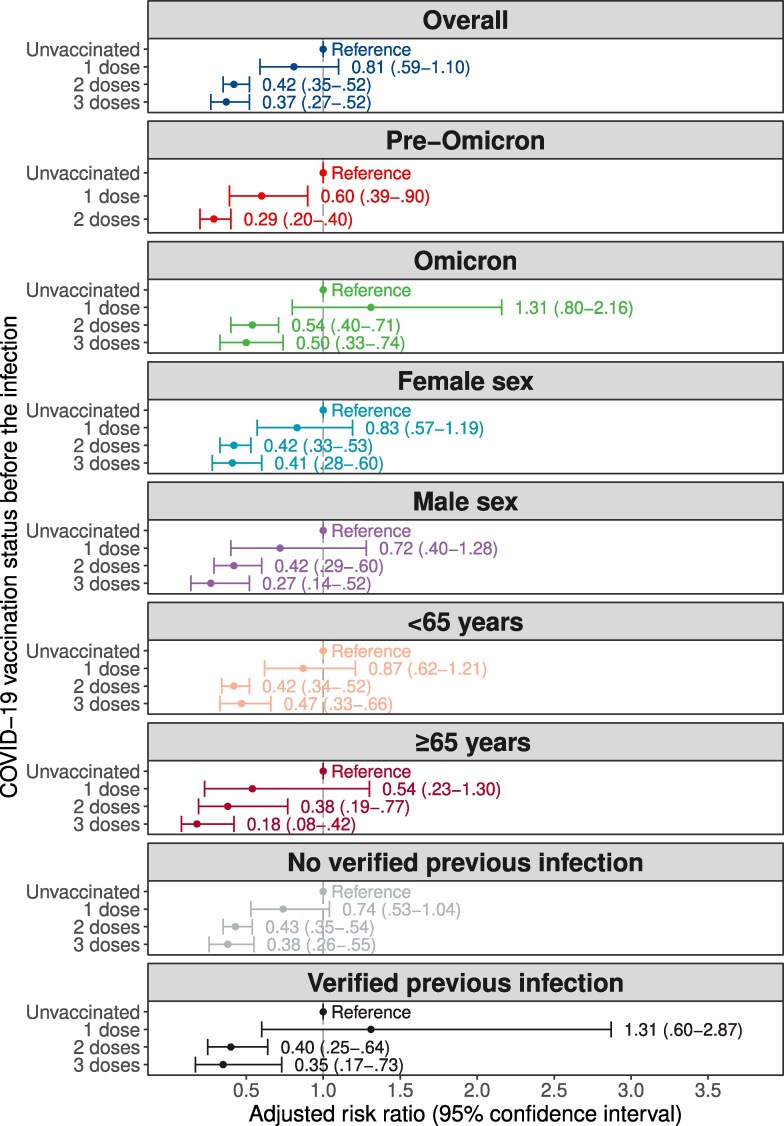
Risk of developing persistent PCC for individuals having received 1, 2, or 3 doses before the SARS-CoV-2 infection compared with unvaccinated individuals. The models for the overall and the pre-Omicron populations were adjusted for age, sex, region of birth, education level, all comorbidities listed in Table 1, previous infection, SARS-CoV-2 variant period, and days since 9 January 2021. The model for the Omicron population was not adjusted for SARS-CoV-2 variant period and days since 9 January 2021. Sex was removed from the subgroup analyses of male and female sex, and previous infections were removed from the subgroup analyses of no verified previous infection and verified previous infection, respectively. All variables used in the models are described in Supplementary Table 1. Because the third dose was not available until September 2021, only 1 and 2 doses were compared with unvaccinated individuals for the pre-Omicron period. Age and days since 9 January 2021 were modelled with restricted cubic splines with 4 knots at the 5th, 35th, 65th, and 95th percentile for individuals experiencing persistent PCC. Abbreviations: COVID-19, coronavirus disease 2019; PCC, post–COVID-19 condition; SARS-CoV-2, severe acute respiratory syndrome coronavirus 2.

Characteristics of the 331 042 included individuals by PCC status at end of follow-up are presented in Table 1. Of the 2546 individuals having a PCC diagnosis any time from 90 days after the PCR test, 33% (n = 852) had persistent PCC. The incidence of persistent PCC was 0.4% (575/136 840) for unvaccinated, 0.3% (32/11 484) for 1 dose, 0.1% (212/157 287) for 2 doses, and 0.1% (33/25 431) for 3 doses. The type of COVID-19 vaccines administered to the study population are presented in Supplementary Table 2. More than two-thirds of all administered doses were of the BNT162b2 vaccine. The adjusted RR model showed reduced risks for developing persistent PCC when vaccinated with 2 or 3 doses compared with unvaccinated individuals: RR was 0.81 (95% CI, .59–1.10) for 1 dose, 0.42 (95% CI, .35–.52) for 2 doses, and 0.37 (95% CI, .27–.52) for 3 doses (Figure 1). A reduced risk of persistent PCC was also observed for individuals having received 2 or 3 doses compared with unvaccinated individuals across all 8 subgroup analyses.

**Table 1.** Characteristics of Study Population by PCC Status at End of Follow-up

Variable	No PCC (n = 328 496)	Nonpersistent PCC^a^ (n = 1694)	Persistent PCC (n = 852)
Female sex, n (%)	178 557 (54.4)	1041 (61.5)	588 (69.0)
Age, y, median (IQR)	41 (32–51)	50 (41–59)	49 (41–58)
Born in Sweden, n (%)^b^	237 402 (72.6)	1048 (61.9)	622 (73.0)
Education level, n (%)^c^			
Primary	39 501 (12.4)	202 (12.1)	94 (11.2)
Secondary	113 615 (35.6)	663 (39.8)	281 (33.4)
Tertiary	165 821 (52.0)	799 (48.0)	467 (55.5)
Comorbidities, n (%)			
Asthma	22 717 (6.9)	243 (14.3)	147 (17.3)
Cancer	3491 (1.1)	33 (1.9)	21 (2.5)
Cardiac/cerebrovascular disease	10 588 (3.2)	149 (8.8)	54 (6.3)
Chronic kidney failure	2614 (0.8)	30 (1.8)	15 (1.8)
Chronic liver disease	1830 (0.6)	14 (0.8)	9 (1.1)
Chronic lung disease	3180 (1.0)	57 (3.4)	29 (3.4)
Diabetes	11 718 (3.6)	159 (9.4)	76 (8.9)
Hypertension	30 156 (9.2)	366 (21.6)	169 (19.8)
Immunosuppression	10 259 (3.1)	112 (6.6)	63 (7.4)
Mental health disorder	86 675 (26.4)	709 (41.9)	422 (49.5)
Neurological disease, including dementia	3339 (1.0)	15 (0.9)	10 (1.2)
Obesity	16 619 (5.1)	175 (10.3)	94 (11.0)
COVID-19 vaccination status, n (%)			
Unvaccinated	135 041 (41.1)	1224 (72.3)	575 (67.5)
1 dose	11 404 (3.5)	48 (2.8)	32 (3.8)
2 doses	156 728 (47.7)	347 (20.5)	212 (24.9)
3 doses	25 323 (7.7)	75 (4.4)	33 (3.9)
Previous SARS-CoV-2 infection	21 606 (6.6)	98 (5.8)	92 (10.8)
SARS-CoV-2 variant, n (%)			
Wild type	14 496 (4.4)	184 (10.9)	81 (9.5)
Alpha	83 902 (25.5)	873 (51.5)	385 (45.2)
Delta	40 426 (12.3)	236 (13.9)	117 (13.7)
Omicron	189 672 (57.7)	401 (23.7)	269 (31.6)
COVID-19 severity, n (%)			
Not hospitalized	320 757 (97.6)	1246 (73.6)	592 (69.5)
Hospitalized	7390 (2.2)	327 (19.3)	153 (18.0)
ICU	349 (0.1)	121 (7.1)	107 (12.6)
Reinfection during follow-up, n (%)	15 312 (4.7)	201 (12.0)	88 (10.3)
No. of health care visits with PCC diagnosis, median (IQR)	NA	1 (1–3)	12 (6–26)
Time from first to last PCC diagnosis, d, median (IQR)	NA	0 (0–64)	414 (302–510)
Reason for end of follow-up, n (%)			
Administrative	322 597 (98.2)	1668 (98.5)	845 (99.2)
Death	1101 (0.3)	9 (0.5)	3 (0.4)
Moving out of Stockholm County	4798 (1.5)	17 (1.0)	4 (0.5)

Abbreviations: COVID-19, coronavirus disease 2019; ICU, intensive care unit; IQR, interquartile range; NA, not applicable; PCC, post–COVID-19 condition; SARS-CoV-2, severe acute respiratory syndrome coronavirus 2.

^a^Defined as having a PCC diagnosis >90 days after the SARS-CoV-2–positive PCR test, but not fulfilling the criteria for persistent PCC as described in the “Methods.”

^b^Data missing for 1510 individuals.

^c^Data missing for 9599 individuals.

## DISCUSSION

We found COVID-19 vaccinations to be associated with a reduced risk of developing persistent PCC among SARS-CoV-2–infected individuals. These effects were consistent both before and after the emergence of the Omicron variant, as well as all other investigated subgroups. It is plausible that part of this effect stems from the protection against severe COVID-19, which has been associated with an increased risk of PCC [2, 3, 5]. Strengths include the use of population-based data sources with high coverage and long follow-up times, including all PCR- and serology-verified infections. An important limitation includes potential outcome misclassification depending on the clinical use of the PCC diagnosis code [2, 3]. Given the broad clinical case definition of PCC and its heterogenous phenotypes, it is possible that PCC might be underdiagnosed as well as overdiagnosed in health care settings as described previously [3, 6]. National survey data and electronic health care record data from the United Kingdom have demonstrated that underestimation is highly plausible [7]. However, we find it unlikely that a major differential outcome misclassification between vaccinated and unvaccinated individuals would yield biased estimates. Collectively, we demonstrate that COVID-19 vaccinations are strongly associated with a reduced risk of persistent PCC, reinforcing the importance of obtaining an adequate protection against COVID-19 and its long-term effects.

## Supplementary Material

jiaf133_Supplementary_Data

## Supplementary Data

Supplementary materials are available at *The Journal of Infectious Diseases* online (http://jid.oxfordjournals.org/). Supplementary materials consist of data provided by the author that are published to benefit the reader. The posted materials are not copyedited. The contents of all supplementary data are the sole responsibility of the authors. Questions or messages regarding errors should be addressed to the author.

## Notes

***Author contributions.*** All authors contributed conceptualization, investigation, methodology, project administration, validation, and review and editing of the manuscript. P. H. performed data curation, formal analysis, and visualization, and wrote the original draft. P. N. contributed funding acquisition, resources, software, and supervision. P. N. and P. H. had full access to all the data in the study and take responsibility for the integrity of the data and the accuracy of the data analysis. All authors have read and approved the manuscript.

***Disclaimer.*** The funders had no role in the design and conduct of the study; collection, management, analysis, and interpretation of the data; preparation, review or approval of the manuscript; and decision to submit the manuscript for publication.

***Data sharing.*** The individual participant data underlying this article were subject to ethical approval and cannot be shared publicly. Data from the deidentified administrative health registry are not freely available due to protection of the personal integrity of the participants.

***Financial support.*** The work was supported by the Swedish Research Council (grant numbers 2021-04809 and 2021-06540); and the European Union Horizon Europe Research and Innovation Programme, EuCARE Project (grant number 101046016).
