# Supplementary material for: The Effect of COVID-19 Vaccination on the Risk of Persistent Post–COVID-19 Condition: Cohort Study
Source: J Infect Dis. 2025 Mar 12;231(5):e941–4. doi: 10.1093/infdis/jiaf133 (PMC12128041; doi:10.1093/infdis/jiaf133)
Supplement: jiaf133_Supplementary_Data [file jiaf133_supplementary_data.docx]

Supplementary material for

**The effect of COVID-19 vaccination on the risk of persistent post COVID-19 condition: Cohort study**

**Authors:** Pontus Hedberg, Suzanne Desirée van der Werff, Pontus Nauclér

Correspondence to [pontus.hedberg@ki.se](mailto:pontus.hedberg@ki.se)

**Table of contents**

| **Content** | **Page** |
| --- | --- |
| Figure S1. Study flow chart | 3 |
| Table S1. Descriptions of study variables | 4-8 |
| Table S2. Types of COVID-19 vaccines administered in the study population | 9 |

**Figure S1. Study flow chart**

**
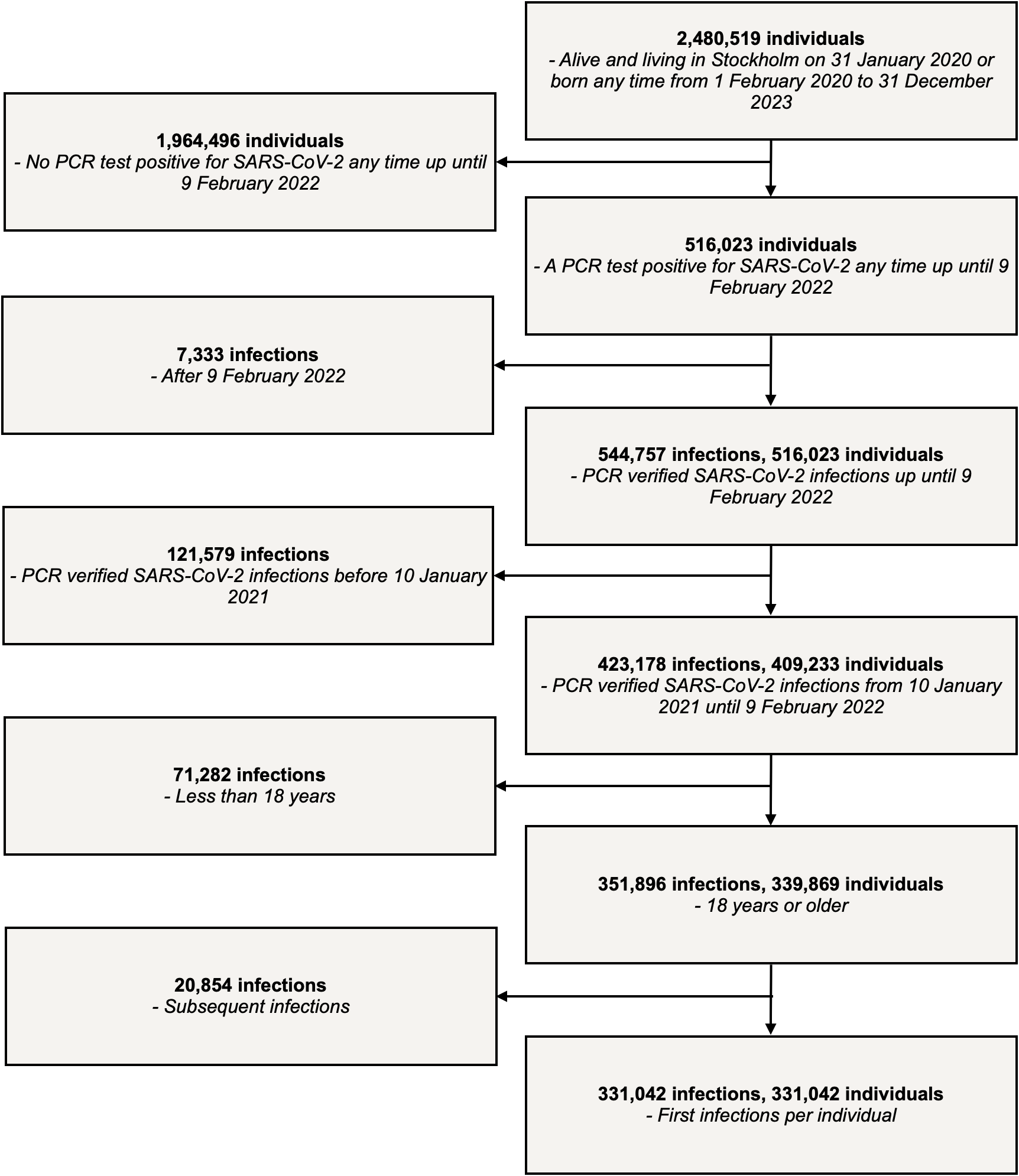
**

**Abbreviations:** PCR=Polymerase chain reaction, SARS-CoV-2=Severe acute respiratory syndrome coronavirus 2

**Table S1. Descriptions of study variables**

| **Variable** | **Data sources** | **Missing data** | **Definition** | **Time period** | **Possible values** |
| --- | --- | --- | --- | --- | --- |
| PCC | VAL, SmiNet | No | No PCC: No PCC diagnosis registered any time from 90 days after the SARS-CoV-2 positive PCR test until the last date of follow-up  Non-persistent PCC: A PCC diagnosis registered any time from 90 to 364 days after the SARS-CoV-2 positive PCR test but not from 365 days after the SARS-CoV-2 positive PCR test until the last date of follow-up  *OR* No PCC diagnosis registered any time from 90 to 364 days after the SARS-CoV-2 positive PCR test but a diagnosis registered from 365 days after the SARS-CoV-2 positive PCR test until the last date of follow-up  Persistent PCC: A PCC diagnosis registered any time from 90 to 364 days after the SARS-CoV-2 positive PCR test and from 365 days after the SARS-CoV-2 positive PCR test until the last date of follow-up | -  -  - | No PCC, Non-persistent PCC, Persistent PCC |
| COVID-19 vaccination status before infection | NVR, SmiNet | No | Number of COVID-19 vaccine doses received any time up until 14 days before the SARS-CoV-2 positive PCR test | 27 December 2020 up until 14 days before the SARS-CoV-2 positive PCR test | Unvaccinated, 1 dose, 2 doses, 3 doses |
| Sex | VAL | No | Biological sex | Birth | Male, Female |
| Age | VAL, SmiNet | No | Age at the date of the SARS-CoV-2 positive PCR test | - | 18-107 years |
| Born in Sweden | Statistics Sweden | Yes, for 1,510 individuals | Region of birth | Birth | Yes, No, Missing |
| Education level | Statistics Sweden | Yes, for 9,599 individuals | Education level in 2019 | End of 2019 | Primary, Secondary, Tertiary, Missing |
| Asthma | VAL, SmiNet | No | ICD-10: J45.X, J46.X | Up to five years before the SARS-CoV-2 positive PCR test | Yes, No |
| Cancer | VAL, SmiNet | No | ICD-10: All codes from C00.X to C97.X besides C44.X, Z51.0, Z51.1  KVÅ: DT107, DT108, DT112, DT116, DT135, DV070, DV071, DV134 | Up to one year before the SARS-CoV-2 positive PCR test  Up to one year before the SARS-CoV-2 positive PCR test | Yes, No |
| Cardiovascular disease | VAL, SmiNet | No | ICD-10: I10.X, I11.X, I12.X, I13.X, I14.X, I15.X (should be registered at least twice during the time period)  ICD-10: I20.X, I21.X, I22.X, I23.X, I24.X, I25.X, I26.X, I27.X, I42.X, I48.X, I50.X, I61.X, I63.X, I64.X | Up to five years before the SARS-CoV-2 positive PCR test  Up to five years before the SARS-CoV-2 positive PCR test | Yes, No |
| Chronic kidney failure | VAL, SmiNet | No | ICD-10: N18.X  ICD-10: Z49.1, Z49.2 (should be registered at least twelve times during the time period)  ICD-10: Z99.2  KVÅ: DR016, DR024 (should be registered at least twelve times during the time period) | Up to five years before the SARS-CoV-2 positive PCR test  One year before the SARS-CoV-2 positive PCR test  One year before the SARS-CoV-2 positive PCR test  One year before the SARS-CoV-2 positive PCR test | Yes, No |
| Chronic liver disease | VAL, SmiNet | No | ICD-10: B18.X, K70.X, K71.7, K72.X, K74.6, K75.X | Up to five years before the SARS-CoV-2 positive PCR test | Yes, No |
| Chronic lung disease (not asthma) | VAL, SmiNet | No | ICD-10: D86.0, D86.2, E84.X, J43.X, J44.X, J46.9, J47.X, J70.3, J84.X, J98.2 | Up to five years before the SARS-CoV-2 positive PCR test | Yes, No |
| Diabetes (type 1 or 2) | VAL, SmiNet | No | ICD-10: E10.X, E11.X | Up to five years before the SARS-CoV-2 positive PCR test | Yes, No |
| Immunocompromised state | VAL, SmiNet | No | ATC: H02AB.X (should be registered at least twice during the time period)  ATC: L01.X  ATC: L04.X  ICD-10: B20.X, B21.X, B22.X, B23.X, B24.X, D57.0, D57.1, D80.X, D81.X, D82.X, D83.X, D84.X, Z94.0, Z94.1, Z94.2, Z94.3, Z94.4, Z94.8  KVÅ: DR04.1, DR04.2, DR04.4, DR04.6, DR04.7  KVÅ: H02AB.X (should be registered at least twice during the time period)  KVÅ: L01.X  KVÅ: L04.X | Up to half a year before the SARS-CoV-2 positive PCR test  Up to one year before the SARS-CoV-2 positive PCR test  Up to half a year before the SARS-CoV-2 positive PCR test  Any time before the SARS-CoV-2 positive PCR test  Up to three years before the SARS-CoV-2 positive PCR test  Up to half a year before the SARS-CoV-2 positive PCR test  Up to one year before the SARS-CoV-2 positive PCR test  Up to half a year before the SARS-CoV-2 positive PCR test | Yes, No |
| Mental health disorder | VAL, SmiNet | No | ICD-10: F20.X-F29.X, F30.X-F39.X, F40.X-F48.X | Up to five years before the SARS-CoV-2 positive PCR test | Yes, No |
| Neurologic disease | VAL, SmiNet | No | ICD-10: F00.X, F01.X, F02.X, F03.X, G10.X, G12.2, G20.X, G30.X, G35.X, G70.X, G71.X, G80.X | Up to five years before the SARS-CoV-2 positive PCR test | Yes, No |
| Obesity | VAL, SmiNet | No | ICD-10: E66.X | Up to five years before the SARS-CoV-2 positive PCR test | Yes, No |
| Previous SARS-CoV-2 infection | SmiNet, Quality Register for SARS-CoV-2 (COVID-19) | No | A positive SARS-CoV-2 serology test up until 14 days before the SARS-CoV-2 positive PCR test, but not after the first COVID-19 vaccine dose *OR*  A previous positive SARS-CoV-2 PCR test (>90 days before the inclusion date in the study) | - | Yes, No |
| SARS-CoV-2 variant | SmiNet | No | Wild-type: SARS-CoV-2 positive PCR test any time from 1 October 2020 to 14 February 2021  Alpha: SARS-CoV-2 positive PCR test any time from 15 February 2021 to 27 June 2021  Delta: SARS-CoV-2 positive PCR test any time from 28 June 2021 to 26 December 2021  Omicron: SARS-CoV-2 positive PCR test any time from 27 December 2021 to 9 February 2022 | SARS-CoV-2 positive PCR test | Wild-type, Alpha, Delta, Omicron |
| COVID-19 severity | VAL, SIR | No | Hospitalized: A hospital admission with a first positive SARS-CoV-2 test any time from 14 days before admission up until date of discharge and a U07.1 or U07.2 ICD-10 code as main or secondary diagnosis at discharge  ICU-treated: Hospitalized in accordance with the definition above plus admission to the ICU any time during this hospitalization.  Not hospitalized: None of the above | SARS-CoV-2 positive PCR test any time from 14 days before admission up until date of discharge | Not hospitalized, hospitalized, ICU-treated |
| Reinfection during follow-up | SmiNet, VAL | No | A positive SARS-CoV-2 PCR test (>90 days after the included infection episode) | >90 days after the included infection episode until date of last follow-up | Yes, No |
| Number of healthcare visits with PCC diagnosis | SmiNet, VAL | No | Number of healthcare visits with a U09.9 diagnosis code registered >90 days after the positive SARS-CoV-2 PCR test up until last date of follow-up | >90 days after the positive SARS-CoV-2 PCR test up until last date of follow-up | 0-156 |
| Time from first to last PCC diagnosis | SmiNet, VAL | No | Number of days from first PCC diagnosis >90 days after the positive SARS-CoV-2 PCR test until the last PCC diagnosis up until last date of follow-up | >90 days after the positive SARS-CoV-2 PCR test until the last PCC diagnosis up until last date of follow-up | 0-570 |
| Reason for end of follow-up | VAL | No | Reason for end of follow-up being the date of death, date of moving out of Stockholm County, or 660 days after the positive SARS-CoV-2 PCR test, whichever occurred first. | Date of death, date of moving out of Stockholm County, or 660 days after the positive SARS-CoV-2 PCR test, whichever occurred first. | Adminstrative, Death, Moving out of Stockholm County |

**Abbreviations:** ATC=Anatomical Therapeutic Chemical, COVID-19=Coronavirus disease 2019, ICD-10=International Classification of Diseases 10^th^ revision; ICU=Intensive care unit, KVÅ=Klassifikation av vårdåtgärder (Swedish for classification of healthcare procedures), PCC=Post COVID-19 condition, SARS-CoV-2=Severe acute respiratory syndrome coronavirus 2, SIR=Swedish Intensive Care Registry, VAL= Stockholm regional healthcare data warehouse

**Table S2. Types of COVID-19 vaccines administered in the study population**

| **Vaccine type** | **First dose (n=194,202)** | **Second dose (n=182,718)** | **Third dose (n=25,431)** |
| --- | --- | --- | --- |
| Ad26.COV2.S, n (%) | 1 (0.0) | 0 (0.0) | 0 (0.0) |
| BNT162b2, n (%) | 157,719 (81.2) | 152,584 (83.5) | 19,467 (76.5) |
| mRNA-1273, n (%) | 26,128 (13.5) | 24,062 (13.2) | 5,963 (23.5) |
| ChAdOx1-S [recombinant], n (%) | 10,354 (5.3) | 6,072 (3.3) | 1 (0.0) |

**Abbreviations:** COVID-19=Coronavirus disease 2019
